# The IL-33/ST2 Axis in Immune Responses Against Parasitic Disease: Potential Therapeutic Applications

**DOI:** 10.3389/fcimb.2020.00153

**Published:** 2020-04-17

**Authors:** Nathan Ryan, Kelvin Anderson, Greta Volpedo, Sanjay Varikuti, Monika Satoskar, Sanika Satoskar, Steve Oghumu

**Affiliations:** ^1^Department of Pathology, The Ohio State University Wexner Medical Center, Columbus, OH, United States; ^2^Division of Anatomy, The Ohio State University Wexner Medical Center, Columbus, OH, United States; ^3^Department of Microbiology, The Ohio State University, Columbus, OH, United States; ^4^Northeast Ohio Medical University, Rootstown, OH, United States

**Keywords:** IL-33, ST2, parasite, innate, immunity

## Abstract

Parasitic infections pose a wide and varying threat globally, impacting over 25% of the global population with many more at risk of infection. These infections are comprised of, but not limited to, toxoplasmosis, malaria, leishmaniasis and any one of a wide variety of helminthic infections. While a great deal is understood about the adaptive immune response to each of these parasites, there remains a need to further elucidate the early innate immune response. Interleukin-33 is being revealed as one of the earliest players in the cytokine milieu responding to parasitic invasion, and as such has been given the name “alarmin.” A nuclear cytokine, interleukin-33 is housed primarily within epithelial and fibroblastic tissues and is released upon cellular damage or death. Evidence has shown that interleukin-33 seems to play a crucial role in priming the immune system toward a strong T helper type 2 immune response, necessary in the clearance of some parasites, while disease exacerbating in the context of others. With the possibility of being a double-edged sword, a great deal remains to be seen in how interleukin-33 and its receptor ST2 are involved in the immune response different parasites elicit, and how those parasites may manipulate or evade this host mechanism. In this review article we compile the current cutting-edge research into the interleukin-33 response to toxoplasmosis, malaria, leishmania, and helminthic infection. Furthermore, we provide insight into directions interleukin-33 research may take in the future, potential immunotherapeutic applications of interleukin-33 modulation and how a better clarity of early innate immune system responses involving interleukin-33/ST2 signaling may be applied in development of much needed treatment options against parasitic invaders.

## Introduction

Interleukin-33 (IL-33) is a member of the IL-1 cytokine superfamily, and plays an important role in innate immunity, inflammatory and autoimmune diseases (Tonacci et al., 2019). Previously it has been presumed that IL-33, through its association with chromatin in the nucleus, acts as a repressor of transcription (Carriere et al., 2007). More recent studies have further elaborated on IL-33 as being a transcriptional regulator of nuclear factor NF- κB where it has demonstrated involvement in the pathogenesis of esophageal squamous cell carcinoma and atherosclerosis, as well as in the activation of endothelial cells (Choi et al., 2012; Buckley et al., 2019; Yue et al., 2019). However, it has been shown in a 2018 study by Travers et al. that IL-33 may have less of a transcriptional regulatory role than was previously thought, and that the role IL-33 plays with chromatin may be post-translational and more involved in controlling the release of nuclear IL-33 (Travers et al., 2018). Additionally, IL-33 has the distinct characteristic of being subject to post-translational modifications that dramatically affect its ability to bind to its receptor, suppression of tumorigenicity 2 (ST2). For example, its affinity to ST2 becomes null after subjection to apoptosis-induced caspases (Cayrol and Girard, 2009; Luthi et al., 2009), while its affinity increases greatly after encountering neutrophil and mast cell-derived serine proteases (Lefrançais et al., 2012, 2014). IL-33 displays high basal expression in endothelial cells and the epithelial cells of many tissues including those of the central nervous system, respiratory, excretory, circulatory, integumentary, and reproductive systems (Yasuoka et al., 2011; Pichery et al., 2012; Cao et al., 2018). It has also been shown that dendritic cells, macrophages, and microglia can produce IL-33 under certain conditions (Pichery et al., 2012; Tjota et al., 2013; Cao et al., 2018). The lack of a secretory sequence and well-defined mechanism for its secretion outside of cell death have led to the designation of IL-33 as an “alarmin,” though some studies suggest it may be released independent of cell death through mechanisms involving mechanical stress, extracellular ATP or active release by macrophages (Molofsky et al., 2015).

IL-33 primarily functions through its receptor ST2 (Liew et al., 2010; Liu et al., 2019). ST2 is a member of the IL-1 receptor superfamily and is found in two spliced isoforms: one soluble and one membrane-bound. The soluble form, sST2, sequesters circulating IL-33, dampening IL-33 signaling. ST2 is the membrane-bound form that participates in signal transduction through myeloid differentiation primary response 88 (MyD88) and nuclear factor NF-κB after binding to its ligand IL-33 (Griesenauer and Paczesny, 2017; Pusceddu et al., 2019). In the innate immune system, ST2 has been shown to be expressed on macrophages, dendritic cells, basophils, eosinophils, mast cells, type 2 innate lymphoid cells (ILC2s), endothelial cells, and neutrophils (Griesenauer and Paczesny, 2017). ST2 signaling has been shown to have pleiotropic effects on these cells including promotion of dendritic cell-mediated activation of ST2^+^ regulatory T cells (Tregs) (Matta et al., 2014), enhancement of lipopolysaccharide response by macrophages (Espinassous et al., 2009), activation of ILC2s (Riedel et al., 2017), promotion of lymphangiogenesis by lymphatic endothelial cells (Han et al., 2017) and induction of eosinophilic chemotaxis, survival and degranulation (Cherry et al., 2008). In the adaptive immune system, ST2 has been shown to be preferentially expressed by Th2 cells, where IL-33 stimulation has been shown to induce the production of IL-4, IL-5 and IL-13 (Schmitz et al., 2005; Paul and Zhu, 2010). IL-33 has also been shown to suppress T helper type 1 (Th1) responses (Rostan et al., 2013; Stier et al., 2019), though some studies have shown that it may potentiate Th1 responses as well in an IL-12 dependent manner (Smithgall et al., 2008; Komai-Koma et al., 2016). A compiled meta-analysis study of the IL-33/ST2 axis has revealed the enormous extent of the complexity and involvement of this signaling pathway, revealing just how much remains to be understood (Pinto et al., 2018).

Interestingly, IL-33/ST2 signaling has been demonstrated to play both pathological and protective roles in various pathologies by exerting pro-inflammatory and anti-inflammatory effects in a context-dependent manner. For example, in the context of obesity, IL-33 was shown to reduce chronic adipose tissue inflammation by promoting the activity of Th2 and alternatively activated macrophage (M2) populations (Miller et al., 2010). However, in the context of cancer, IL-33 aids in tumor immune evasion by upregulating the activity, survival and expansion of myeloid derived suppressor cells via ST2 signaling, which was reduced in ST2^−/−^ mice (Xiao et al., 2016). Recent discoveries have further suggested that IL-33 can induce the proliferation, survival, and metastasis of cancer cells (Allegra et al., 2019; Gorbacheva and Mitkin, 2019). IL-33 is also known to exacerbate asthmatic and allergic inflammation in the skin, GI tract and lungs through the ST2-mediated activation of basophils, eosinophils, mast cells, dendritic cells, macrophages and ILC2s by promoting the chemo-attraction of Th2 cells and the production of Th2-associated cytokines by various cell types (Louten et al., 2011; Bartemes et al., 2012; Sjoberg et al., 2017; Chan et al., 2019). In the context of infectious disease, many studies have further shown that IL-33/ST2 activity displays context-dependent protection and exacerbation of infection dependent on multiple factors, including the infectious agent, cellular microenvironment, and affected organs. IL-33 has been demonstrated in viral infection to promote a protective immune response through enhanced CD8^+^ T cell responses (Bonilla et al., 2012). Interestingly, viral infection within lung tissue has shown a necessity for ST2^+^ ILC accumulation in influenza recovery (Monticelli et al., 2011). However, contrary to this point, it has been observed that over-expression of IL-33 may be associated with COPD (Byers et al., 2013). IL-33/ST2 signaling has further been demonstrated to be of positive benefit in innate immunity at the site of the skin, where its expression activates downstream production of antimicrobial reactive oxygen and nitrogen species (Li et al., 2014). Pathologies associated with bacterial infections including pediatric asthma and *Staphylococcus aureus* induced septic arthritis, however, have been found to respond negatively with expression of IL-33 or ST2 (Hentschke et al., 2017; Staurengo-Ferrari et al., 2018). IL-33 involved mechanisms have also been demonstrated to show divergent roles in the resistance to different fungal infections, where it has been noted to be protective in the context of candidiasis but hinders clearance of *Aspergillus fumigatus* infection (Park et al., 2016; Garth et al., 2017). Within this review, we will be focusing on IL-33/ST2 signaling within the context specifically of parasitic infection.

Parasitic diseases affect a significant percentage of the world's populations, with billions being infected or at risk of infection (Hay et al., 2004; Torgerson et al., 2015; Short et al., 2017; Jourdan et al., 2018), with more and more becoming susceptible due to factors such as climate change, increasing population density, loss of biodiversity, habitat restriction and overall ecological remodeling (Cable et al., 2017; Short et al., 2017). Despite recent improvements in the infection and mortality rates of parasitic diseases like malaria (WHO, 2019), issues such as drug resistance by both parasites and vectors pose a significant threat (Sibley and Hunt, 2003; Vanaerschot et al., 2014; Bushman et al., 2016; Alout et al., 2017). Host-directed approaches toward therapies displays significant promise, though further research is needed to make their application viable (Varikuti et al., 2018). Research investigating IL-33/ST2's role in parasitic infection shows that its modulation may demonstrate a viable treatment strategy, though due to the varying nature in IL-33/ST2 signaling in the host immune response, there is a need for further research on the topic. While the IL-33/ST2 signaling axis has been researched in the context of many systems and diseases, the roles of such findings in the context of parasitic disease have not been exhaustively compiled. In this review, we explore IL-33/ST2 signaling of the innate immune system's response and provide insight into its role during parasitic infections caused by *Toxoplasma, Plasmodium, Leishmania*, and helminths.

## Toxoplasma

*Toxoplasma gondii*, the causative agent of the neglected parasitic disease toxoplasmosis, is an obligate intracellular protozoan capable of infecting most types of mammalian cells. Toxoplasmosis affects 25–30% of humans worldwide and is usually asymptomatic in immunocompetent individuals, but can become a life threatening condition in immunocompromised patients as well as in developing fetuses (Delgado Betancourt et al., 2019; Lima and Lodoen, 2019). Toxoplasmosis can be transmitted via consumption of contaminated food, zoonotically or congenitally (Delgado Betancourt et al., 2019). Oocysts are excreted in the feces of infected animals and can be consumed by other animals. In the new host, oocysts release sporozoites, which can then differentiate into bradyzoites and tachyzoites. Tachyzoites can form cysts in various organs, which predator animals may consume. After ingestion, cysts release bradyzoites, which can then convert back into the fast growing tachyzoites and infect the surrounding tissue (Delgado Betancourt et al., 2019). Once an individual becomes infected, the parasite can disseminate through the bloodstream and establish a chronic infection in different organs. The three canonical types of toxoplasmosis are cerebral, lymphatic and ocular (Lima and Lodoen, 2019). After the entry of infected tachyzoids into the host intestine, a rapid recruitment of neutrophils occurs, followed by the action of other immune cells such as macrophages and dendritic cells. These cells elicit a strong inflammatory response characterized by the production of IL-12 and interferon-gamma (IFN-γ), inducers of a protective Th1-type immunity (Khan et al., 2019; Ryffel et al., 2019). This T cell-mediated immunity is crucial for resolving acute infection and controlling chronic disease. *Toxoplasma gondii* is also able to inhibit apoptotic pathways in infected mammalian cells (Lima and Lodoen, 2019).

While a pro-inflammatory response is important for controlling the parasites, a Th2 response is necessary to prevent pathology and tissue damage caused by over-active Th1 responses. A balance between Th1 and Th2 responses is crucial for controlling toxoplasmosis. The Th2 response can be amplified by several cytokines, including IL-33. IL-33 signaling through T1/ST2 was shown to be required for controlling *Toxoplasma* infection in the brain and preventing the development of encephalitis. T1/ST2^−/−^ BALB/c mice infected with *Toxoplasma* showed increased pathology and parasitic burdens and had higher levels of *Nos2, Ifng* and *Tnf* mRNA transcripts in their brains compared to T1/ST2^+/+^ mice (Jones et al., 2010).

In the eye, an immune-privileged site, the immune response to toxoplasmosis is different compared to other organs. Immune-mediated inflammation is reduced in the eye, but the preservation of immune privilege is dependent upon immune-suppressive responses (Tong and Lu, 2015). Ocular toxoplasmosis can cause vision-threatening complications depending on the levels of inflammation. The action of IL-33 is important to control inflammation and pathology in the eye (Zhang et al., 2019). Kunming mice infected intraocularly with *Toxoplasma* showed increased numbers of IL-33 positive cells as well as higher levels of IL-33 and ST2 mRNA transcripts in the eyes and cervical lymph nodes. Additionally, there was a significant correlation between the levels of IL-13 and ST2 and also the levels of IL-4 and ST2, suggesting that IL-33 signaling may be involved in the immunopathology of ocular toxoplasmosis (Tong and Lu, 2015). Higher levels of *Il33* mRNA transcripts were also found in the eyes of *Toxoplasma*-infected susceptible C57BL/6 mice compared to resistant BALB/c mice; however, it was not clear whether this or other cytokines were responsible for the ocular pathology seen in C57BL/6 mice (Zhang et al., 2019).

In an oral model of *Toxoplasma* infection, absence of IL-33 receptor/ST2 attenuated neutrophilic inflammation and ileitis in susceptible C57BL/6 mice and enhanced their survival. These effects are mediated by the increased expression of IL-22, a protective cytokine of the IL-10 family released mainly by dendritic cells and Th17 cells. Blockade of ST2 via neutralizing anti-ST2 antibodies conferred partial protection, while blockade of IL-22 abrogated this protection. These findings show that IL-33 plays a dual role in inflammation (Ryffel et al., 2019).

A delicate balance between the pro and anti-inflammatory response is crucial for controlling toxoplasmosis. While inflammation is needed to eliminate the parasite and the infected cells, an anti-inflammatory response is necessary to limit tissue damage. IL-33 signaling through the ST2 receptor has been shown to play a dual role in inflammation and can therefore have different effects on toxoplasmosis depending on the tissue. For instance, in the brain and eye IL-33 controls immunopathology and is instrumental for disease resolution, while in an oral model of toxoplasmosis, the action of this cytokine is detrimental to murine survival. More studies are needed to fully determine the complex role of IL-33/ST2 in different infected tissues and stages of toxoplasmosis. A better understanding of this signaling pathway could aid the discovery of novel immunomodulatory therapies against toxoplasmosis.

## Plasmodium

Malaria is widely known as the parasitic disease with the most damaging global impact, with an estimated 228 million cases occurring in 2018, and to which ~405,000 deaths can be directly attributed, most of whom are children under 5 in Sub-Saharan Africa. While these numbers are an improvement from recent years, they remain dangerously high (WHO, 2019). Malaria is a mosquito-borne illness spread by *Anopheles* mosquitoes that is known to be caused by six species of protozoans from the genus *Plasmodium*, though the most virulent and prevalent is *Plasmodium falciparum* (Milner, 2018; WHO, 2019). Upon entry to the host through mosquito saliva, *Plasmodium* sporozoites enter the bloodstream and invade hepatocytes, where they germinate via schizogony into merozoites in what is known as the liver stage of infection. The subsequent blood stage of infection is initiated when the merozoites release from the hepatocytes into the bloodstream to invade erythrocytes, where they feed as trophozoites and further replicate via schizogony before liberating from the erythrocytes, continuing the blood stage of infection (Miller et al., 2002). Malaria leads to system-wide symptoms, the most severe of which occur in the form of hypoglycemic acidosis, anemia, renal failure, respiratory failure and cerebral malaria, which have been implicated to occur in the blood stage as a result of the sequestration of infected erythrocytes in microvasculature, the rupture of erythrocytes, and the systemic burden of circulating parasites (Miller et al., 2002, 2013; Bartoloni and Zammarchi, 2012). Interestingly, evidence has mounted to implicate the immune system in playing both protective and pathological roles in malaria symptomology, with context-dependent inflammatory responses helping the host by reducing parasitemia on one hand, but hurting the host by contributing to severe symptomology and sequelae on the other (Miller et al., 2002; Gowda and Wu, 2018; Pais and Penha-Goncalves, 2018).

Emerging evidence has implicated IL-33 and ST2 as having significant influence on the severity of symptoms in malarial infection. The role of IL-33/ST2 axis in early onset infection remains unclear. Previously, a study using intradermal injection of *Plasmodium berghei* sporozoites into C57BL/6 mice showed no differences in IL-33 expression via RTqPCR within the draining lymph nodes compared to uninfected controls 1.5 h after infection (Mac-Daniel et al., 2014). However, it should be noted that IL-33 is constitutively expressed and maintained at high basal levels in endothelial and epithelial cells, and can be released when these cells are damaged (Moussion et al., 2008). Thus IL-33 may not need to be further induced to be biologically active in early malarial infection, though any role it may play at this stage is yet to be elucidated. Moreover, IL-33 is known to play a role in the activity of tissue-resident immune cells, including mast cells and ILC2s. Specifically, IL-33 has been shown to potentiate mast cell activity (Komai-Koma et al., 2012; Joulia et al., 2017), while mast cell degranulation has been separately shown to correlate with *P. falciparum* severity (Wilainam et al., 2015); though it also has been demonstrated to promote the expansion of ST2^+^ ILC2s in the skin (Salimi et al., 2013), a cell type whose systemic expansion is shown to correlate with improved protection against cerebral malaria (Besnard et al., 2015). Ultimately, IL-33's importance in these processes remains speculative and associative in the context of malaria, providing rationale to further explore and identify the mechanisms and activities of IL-33 during the initial malarial infection.

Interestingly, IL-33 has been associated with severe respiratory symptoms during malaria infection. A study analyzing histological samples taken from the lungs of Southeast Asian patients who had died from severe *P. falciparum* infection showed a significant accumulation of IL-33 in their bronchioles, which correlated with severity of pathological pulmonary remodeling and inflammatory lymphocyte, monocyte, and neutrophil recruitment into the lungs (Ampawong et al., 2015). Likewise, a study using a *P. berghei* ANKA (PbA) model of infection on C57BL/6 mice showed that malaria-induced acute respiratory distress was alleviated by dexamethasone treatment, which coincided with decreased levels of serum IL-33 (dos Santos Ortolan et al., 2018). These findings are especially significant considering the tendency for IL-33 transgenic mice to develop spontaneous pulmonary inflammation (Zhiguang et al., 2010), though further research is needed to elucidate the mechanisms by which IL-33 contributes to *Plasmodium*-induced pulmonary distress.

Further evidence for a role of IL-33 in malarial infection was demonstrated in a study by Ayimba et al. assessing pediatric patients under the age of 5 in central Togo, which found that IL-33 was significantly elevated in the plasma of patients with severe *P. falciparum* infection (classified by a parasite load of 250,000/μL and/or a hemoglobin concentration of 5g/dL) compared to infection-free controls, with the authors calling for further exploration of IL-33's role in cerebral malaria (Ayimba et al., 2011). Cerebral malaria is a severe complication of *P. falciparum* infection marked by coma, seizures, and neurological abnormalities. Furthermore, survivors of cerebral malaria typically demonstrate debilitating sequelae manifesting from neurological damage including cognitive, motor and behavioral deficits (Idro et al., 2005, 2010). Currently, the underlying mechanism of cerebral malaria is poorly understood, though inflammatory cytokines appear to play a major role (Armah et al., 2005; Idro et al., 2010). The logic behind Ayimba et al.'s call for investigation into IL-33 was due in part to constitutive expression of IL-33 in the central nervous system by endothelial cells and astrocytes, and the observation that ST2^+^ microglia demonstrate pro-inflammatory effects via upregulation of inflammatory cytokines and chemokines, nitric oxide and microglial phagocytosis (Yasuoka et al., 2011; Fairlie-Clarke et al., 2018).

Per the suggestion of Ayimba et al. the roles of IL-33 and its receptor ST2 have been investigated mechanistically, and the results of these studies have implicated them as major players in cerebral malaria. Surprisingly, IL-33/ST2 was shown to have potential therapeutic properties via direct administration of IL-33 as well as serving as an etiological component of cerebral malaria pathogenesis. One study using a blood-stage PbA model of infection on C57BL/6 mice found that administration of IL-33 attenuated the development of experimental cerebral malaria, with its therapeutic effect being attributed to induction of M2 polarization, reduction of inflammatory mediators and expansion of CD45^+^ST2^+^ICOS^+^ ILC2s and Tregs (Besnard et al., 2015). Specifically, this study found that IL-33 induced ILC2s in promoting M2 activity *in vitro* and *in vivo*, which could promote Treg expansion *in vitro*. This group demonstrated using PbA-infected Treg-depleted mice receiving IL-33 or no treatment displayed significant cerebral malaria compared to mice receiving IL-33 with normal Treg function. This downregulated Treg activity resulted in with increases in serum levels of IL-12, IFN-γ as well as IFN-γ and granzyme b expression by CD8^+^ cells compared to IL-33 treated Treg competent mice. Taken together, this demonstrates that IL-33 mediated Treg function is important in preventing cerebral malaria, though the exact mechanisms of which remains to be elucidated. Another study using the same model of infection corroborated these findings, showing that IL-33 was downregulated in experimental cerebral malaria and that administration improved the efficacy of the anti-malarial drugs artesunate and chloroquine, though no therapeutic effect was elicited by IL-33 alone when administered in the context of experimental cerebral malaria. Mechanistically, this study found that IL-33 administration in concomitance with artesunate and chloroquine led to decreased IL-1β production and NLRP3 inflammasome formation in the brain compared to mice treated with the anti-malarial drugs alone (Strangward et al., 2018). This finding is particularly significant when taking into account the NLRP3 inflammasome's etiological role in neuroinflammatory diseases (Song et al., 2017) and IL-1β's association with fatal cerebral malaria (Maneerat et al., 1999).

Paradoxically, another study using the PbA model of malaria showed antithetical results, with ST2-deficient mice showing significantly reduced experimental cerebral malaria symptoms. Specifically, this study found reduced cerebral inflammation and decreased pathological migration of T-cells to the brain in ST2-deficient mice via downregulation of ICAM-1 correlating with a reduction in LT-α, though no differences were observed in systemic and pulmonary inflammation (Palomo et al., 2015). Interestingly, this lack of neural T cell infiltration in ST2^−/−^mice occurred independently of the CXCR3-associated chemokines CXCL9/10 expression and did not correlate with any changes in expression of granzyme b, IFN-γ, or TNF-α in lysates of the whole brain (Palomo et al., 2015). This same group in a later study demonstrated that ST2^−/−^ mice showed resistance to cognitive defects resulting from PbA infection and improved survival compared to WT mice. The improved prognosis observed in ST2^−/−^ mice was attributed to preservation of neurogenesis pathways and reduced inflammatory cytokine production by hippocampal glial cells, including IL-1β, which they found in turn to stimulate IL-33 production by oligodendrocytes (Reverchon et al., 2017). IL-33 produced by oligodendrocytes could further induce IL-1β and inflammatory activity in glial cells, creating a pathological feedback contributing to cerebral malaria. Surprisingly, within the hippocampus, they found increased levels of CXCL9 and CXCL10 as well as IFN-γ, IL-6 and TNF-α, contrary to previous showing no difference in these chemokines in the whole brain (Reverchon et al., 2017). Additionally supporting a pathological role of IL-33 in malaria, a study utilizing a *Plasmodium chaubadi* model of infection on BALB/c mice showed that deficiency of ST2 reduced mortality, hepatocyte damage and inflammatory cytokine production (Seki et al., 2018). Further complicating the matter, another group found that IL-33 was not upregulated in the brain following PbA infection and that IL-33^−/−^ showed a similar survival and parasitemia to wild-type mice; though IL-33 was found to be decreased in the liver and increased in both the lungs and spleen in wild-type mice after infection (Shibui et al., 2016).

**Figure 1:**
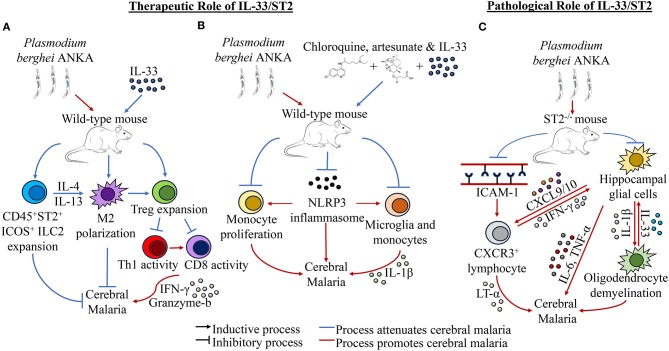
Effects of IL-33 in the context of cerebral malaria. **(A)** IL-33 treatment in the context of *Plasmodium berghei* ANKA (PbA) has been shown to induce the expansion of CD45^+^ ST2^+^ICOS^+^ ILC2s and Tregs, as well as induce M2 polarization. Specifically, ILC2s elicit M2 expansion, while M2s can promote Treg activity. Tregs themselves reduce cerebral malaria by downregulating the activity of Th1 and CD8^+^ cells, which induce neurological inflammation and cerebral malaria through effectors such as IFN-γ and granzyme b. **(B)** IL-33 in concomitance with the antimalarial drugs artesunate and chloroquine has been shown to improve the outcome of PbA infection by reducing cerebral malaria. It has been observed specifically that addition of IL-33 to these therapies resulted in decreased NLRP3 inflammasome formation, monocyte expansion, and IL-1β production by monocytes and microglia. **(C)** ST2^−/−^ mice have been shown to have reduced cerebral malarial cognitive function and improved survival in PbA infection. This reduction has been attributed to reductions in ICAM-1 expression and CXCR3^+^ lymphocyte populations in the brain, which corresponded with lower LT-α levels in the brain, though other inflammatory cytokines expression has not been shown to be significantly affected. Separately, it has been shown that hippocampal glial cells are stimulated to produce IL-1β, which in turn stimulates oligodendrocytes to increase IL-33 expression. Upregulated IL-33 in turn induces more inflammatory activity in hippocampal glial cells, which creates a positive feedback loop that leads to hippocampal demyelination and increased inflammation, resulting in cerebral malaria morbidity and cognitive dysfunction. Additionally, increased levels of CXCL9 and CXCL10 found in the hippocampus could recruit Th1 cells to the site, further exacerbating cerebral malaria through inflammatory induction of microglia through IFN-γ.

Given the pleiotropic nature of IL-33, these findings when taken together could suggest that IL-33 activity on ST2^+^ cells may contribute to the development of severe cerebral malaria, while IL-33 activity may induce a therapeutic effect outside of the IL-33/ST2 axis. Another school of thought is that cell-specific ST2 activity may play a contributory role in the pathogenesis of cerebral malaria in a differentially IL-33 dependent or IL-33 independent manner. Nonetheless, these ostensibly diametric findings demonstrate a gap in our understanding of the role of IL-33 in malarial infection, thus highlighting the need for more research to bolster our understanding of this complicated cytokine in the context of cerebral malaria. The effects of IL-33 in the context of cerebral malaria is summarized in Figure 1.

## Leishmania

Leishmaniasis is the second-largest neglected tropical disease and recent estimates by the CDC approximate that upwards of 1.2 million new cases will be diagnosed this year (https://www.cdc.gov/parasites/leishmaniasis/epi.html). Leishmaniasis is caused by more than 20 different species of protozoan parasite belonging to the genus *Leishmania* (http://www.who.int/leishmaniasis/en/). *Leishmania* is more prevalent in countries of tropical and subtropical regions, especially in areas of lower socioeconomic position. This disease is classified into three types based upon the type of parasite infection and disease outcome. The most common form is cutaneous leishmaniasis (CL), which causes painless localized skin lesions leading to severe tissue damage and disfigurement without treatment (Varikuti et al., 2017). CL is caused by *L. major, L. tropica* and *L. aethiopica* in the old world (Asia and Africa) and *L. mexicana* and *L. braziliensis* in the New World (Central and South America) (Oghumu et al., 2015). The most severe form of the disease is Visceral Leishmaniasis (VL) which is mainly caused by *L. donovani, L. chagasi* and *L. infantum* (McGwire and Satoskar, 2014). VL is characterized by the quick progression of parasites into the liver, spleen and bone marrow resulting in anemia, weight loss and hepatosplenomegaly and ultimately leading to the death of the host if left untreated (de Freitas et al., 2016).

The protective host immunity toward leishmaniasis primarily depends on the type of infection. Generation of an appropriate Th1 immune response, such as the production of IFN-γ and activation of phagocytic cells, is critical to host immunity against both CL and VL, as they lead to the production of reactive nitrogen species which directly cause the death of intracellular parasites (Oghumu et al., 2015; Terrazas et al., 2017; Varikuti et al., 2019). On the other hand, Th2 responses characterized by the production of IL-4 and IL-10 are known to exacerbate CL (Oghumu et al., 2010). In contrast to CL, the Th2-associated cytokines IL-4 and IL-13 are shown to play a protective role in VL by inducing the formation of mature hepatic granulomas and clearance of the parasites (Stäger et al., 2003; McFarlane et al., 2011). Since IL-33 is involved in the activation of Th2 cells, as well as Th1 and CD8^+^ cells, a rationale exists for exploring its role in both VL and CL.

Recent studies have detected significantly elevated levels of IL-33 in the serum of both VL patients and mice infected with VL caused by *L. donovani*. In addition, higher proportions of IL-33^+^ cells were also detected in liver biopsy specimens from VL patients who competed in the healthy tissues (Rostan et al., 2013). It has been also shown that ST2 deficient mice can control hepatic parasitic burdens and have reduced hepatomegaly and splenomegaly resulting in protection against experimental VL caused by *L. Infantum* (Khalid et al., 2017). Additionally, lack of ST2 also resulted in increased IFN-γ expression by both CD4^+^ T and CD8^+^ T cells suggesting depletion of ST2 possibly leads to a shift of immune responses toward Th1-polarization. A recent study has shown that increased levels of IL-33 were detected in malnourished human patients suffering from VL, suggesting that this may also impact Th1 immune responses and contribution of inflammation (Takele et al., 2016).

Our current understanding of the involvement of IL-33 and ST2 signaling in *Leishmania* infection suggest an upregulation of these signaling molecules results in deficient Th1 cellular immune responses. IL-33 activation of a Th2 cellular immune response would be detrimental in patients suffering from leishmaniasis, and inhibition of this signaling has been demonstrated to abrogate infection within the liver of VL. While it is presently understood that IL-33 is modulated during *Leishmania* infection, the research available is scarce and a great deal remains to be elucidated.

## Helminths

Helminth infections in humans are the result of infestation by a wide variety of nematodes, cestodes, trematodes and acanthocephalans (Mathison and Pritt, 2018), the most common of which are the soil-transmitted helminths *Ascaris lumbricoides, Trichuris trichuria, Necator americanus* and *Ancylostoma duodenale* as well as members of the genus *Schistosoma*, the causative agent of schistosomiasis. According to most recent estimates by the World Health Organization and other epidemiological studies about 1.5 billion or 24% of the global population and another 207 million are infected with either soil-transmitted helminths or *Schistosoma*, respectively (Hajissa et al., 2018; Jourdan et al., 2018). With such widespread transmission any development in the treatment or prevention of helminth infection would have significant global impact, and a better understanding of the initial response of the host to helminth infection will guide research toward these critical developments.

While helminths are known to rarely infect ectopic niches in the human host, they are typically found harbored within the intestines. Expulsion of helminths from the host is understood to require a strong type-2 immune response utilizing host ILC2s, M2s, mast cells, eosinophils and ultimately CD4^+^ Th2 cells (Grencis, 2015). Additionally, due to their location within the gut, helminths elicit a cytokine response from intestinal epithelial cells as well, including epithelial tuft cells which have been shown to respond to the presence of helminths by expanding and releasing the Th2 regulatory cytokine IL-25 (Gerbe et al., 2016). It is the end goal of the immune response to helminth parasites to induce goblet cell hyperplasia and increased mucin production which will result in the removal of the resident parasites (Marillier et al., 2008).

IL-33 is constitutively expressed by epithelial barrier cells, especially those lining the intestine, and is released upon cellular damage or death caused by helminth activity. Andronicos et al. demonstrated this showing increased IL-33 mRNA expression in epithelial cells using an *in vitro* human epithelial cell-helminth co-culture system (Andronicos et al., 2012). Once released, Il-33 is free to interact with its receptor, ST2, or to be cleaved into a more active, mature form, by local neutrophils or mast cells prior to interacting with its ligand (Lefrançais et al., 2012, 2014). ST2, originally found to be expressed exclusively by Th2 cells, has since been found to be expressed by a variety of other leukocytes including Tregs, ILC2s, M2s, mast cells, eosinophils, basophils and natural killer cells (Moritz et al., 1998; Xu et al., 1998; Brett Cherry et al., 2008; Smithgall et al., 2008; Suzukawa et al., 2008; Price et al., 2010; Schiering et al., 2014). As an alarmin, IL-33 proceeds to function to promote an early shift toward a type 2 immune response, protective in the context of helminth infection. Upon interaction with its ligands, especially those expressed by ILC2s and mast cells, activated cells respond by considerably enhancing production of type 2 cytokines IL-5 and IL-13 (Henry et al., 2017). While not its only target, IL-33 is known to interact especially well with those ILC2s (Gorbacheva and Mitkin, 2019). These cytokines are critical in the expulsion of intestinal helminths, as they cause the increased production of mucin in the intestine as well as induce goblet cell hyperplasia, inhibiting the ability of the worms to adhere to the gut lumen and allowing the host to rid themselves of the invading parasites (Hasnain et al., 2011).

Beginning with the damage of epithelial cells and their subsequent IL-33 release in response to helminth infection, cells of the host immune system respond in a type-2 manner which has been shown to be necessary in a number of recent human studies and animal models. A 2018 study using a *Hymenolepis diminuta* mouse model of helminth infection demonstrated that mast cell deficient mice required a longer time frame to completely expel infecting parasites (González et al., 2018). Parasites which remained and were unable to be expelled were also found to be larger in size than those removed from mice possessing mast cells. Furthermore, they found decreased expression of TSLP, IL-25 and IL-33 in mice lacking mast cells. Inhibition of mast cell function *in vivo* has also been linked to an overall reduction in IL-33, as well as IL-25 and TSLP both in the context of helminth infection as well as without (Hepworth et al., 2012). Utilizing mice deficient in IL-33, Yasuda et al. has further shown that IL-33 plays an important role in sufficient activation and recruitment of mast cells (Yasuda et al., 2012). Ultimately, mice with ineffective mast cells are unable to prime an effective type 2 immune response regulated by IL-33, IL-25 and TSLP in response to helminth infection and are unable to clear the parasites.

Eosinophils, largely associated with the response to helminth infection and allergies, have also been proven to possess strong anti-helminthic effects mediated by IL-33. In a model in which mice are pre-sensitized to an allergen, Gazzinelli-Guimaraes et al. show that subsequent infection with *Ascaris lumbricoides* presents with abrogated effect. This helminth preventive effect was associated with significantly increased IL-4, IL-13, and IL-33 in only those mice pre-sensitized with the allergen (Gazzinelli-Guimaraes et al., 2019). Detectable levels of eosinophil peroxidase as well as IL-5 and IL-13 are suggestive of the presence of an activated cohort of eosinophils in sensitized mice. Interestingly, eosinophil deficient ΔdblGATA mice lose the protective ability to thwart a helminth infestation post-sensitization, demonstrating the necessity of these innate immune cells in combatting helminth infection. Using IL-33^−/−^ mice, Yasuda et al. demonstrated a critical role for IL-33 in eosinophil recruitment and associated goblet cell hyperplasia (Yasuda et al., 2012). Looking at mRNA transcripts for eosinophilic recruitment and activation cytokine IL-5, IL-13, and CCL11 in IL-33^−/−^ mice they found significantly decreased quantities of each cytokine, suggesting a possible involvement of IL-33 in the production of these eosinophil associated cytokines.

Finally, it has been shown that, in association with enhanced eosinophilia, IL-33 is also required for the appropriate accumulation of helminth-protective ILC2s (Yasuda et al., 2012, 2018). Using mice deficient in IL-33 this group shows that without IL-33 ILC2s are unable to effectively accumulate, resulting in increased parasite burden associated with a decrease in mRNA transcripts for Th2 cytokines. Interestingly, it was also demonstrated that the innate immune system independent of adaptive immunity is sufficient to induce goblet cell hyperplasia, as RAG-2^−/−^ mice deficient in T and B-cells still exhibit hyperplastic goblet cells when provided with exogenous IL-33 (Kondo et al., 2008). Taken together, these results paint a clear picture of the critical role the innate immune system plays in response to helminth infection that is, at least in part, mediated through IL-33 released at first contact by invading parasites.

**Figure 2:**
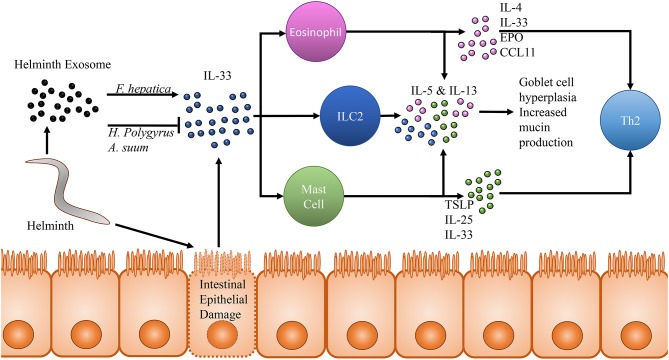
Effects of IL-33 in the context of helminth infection. Movement and other activity by helminths within the intestinal lumen cause damage and lysis of intestinal epithelial cells. These epithelial cell in turn release pre-formed IL-33, which acts upon many cells of the innate immune system through its receptor ST2. Notably among these cells populations are eosinophils, type 2 innate lymphoid cells and mast cells. All three of these cell populations release IL-5 and IL-13 in response to IL-33 stimulation, resulting in goblet cell hyperplasia and increased mucin production. This effector response has been shown to be capable of clearing helminth infection even in RAG^−/−^ mice who are incapable of generating a Th2 adaptive immune response. In addition to IL-5 and IL-13, studies focusing on eosinophils and IL-33 in response to helminth infection have also detected increased IL-4, IL-33, eosinophil peroxidase and CCL11 suggesting increased eosinophil accumulation and activity in response to IL-33 as well as a shift toward a Th2 immune response. Mast cells have been demonstrated to release thymic stromal lymphopoietin, IL-25 and IL-33 in response to IL-33 promoting a shift toward a protective Th2 immune response. In response to this defense, two species of helminth, *Heligmosomoides polygyrus* and *Ascaris suum* have been observed to release an exosome capable of inhibiting IL-33 in the host and dampening the subsequent helminth protective immune response. Interestingly, one helminth, *Fasciola hepatica*, possesses an exosome which has been observed to seemingly counter-intuitively upregulate IL-33 by the host, presenting just one example of the different physiological responses each helminth may induce and further expounding upon the importance of studying each helminth as an individual which cannot be easily generalized based on other helminth research.

While great attention is being paid to host innate immune cell specific mechanisms of subduing helminths, it is also being uncovered that helminths have evolved such that they possess a number of ways to modulate or evade the host immune system in an IL-33/ST2 dependent manner. The mouse pathogenic helminth *Heligmosomoides polygyrus* was recently found to secrete vesicles in the intestinal lumen of mice that are rich in inhibitory miRNAs homologous with mammalian exosome proteins (Buck et al., 2014). RT-qPCR analysis of mice treated with *H. polygyrus* derived exosomes found a downregulation in transcripts for *Il33r* coding for the ST2 protein. Interestingly, this same group previously showed that excretory products taken from *H. polygyrus* inhibits initial release of IL-33 *in vivo* with a subsequent inability for ILC2s and eosinophils to aggregate with an associated down regulation in IL-4, IL-5, and IL-13 (McSorley et al., 2014). Additionally, *H. polygyrus* vesicles have been demonstrated to be taken up by host macrophages, resulting in a downregulation of ST2, inhibiting their ability to function effectively as protective M2s (Coakley et al., 2017). Extracellular vesicles released by the porcine helminth *Ascaris suum* have also been identified as possessing numerous miRNA transcripts which likely target IL-13, IL-25 and IL-33 (Hansen et al., 2019). However, it is important to note that in the context of helminth infection, host-parasite interactions may vary widely dependent upon the specific helminth. Mice treated with *Fasciola hepatica* vesicles demonstrate an upregulation in IL-5 and IL-33 expression, highlighting the variety in immunomodulatory strategies of different helminths (Finlay et al., 2016). Despite mounting evidence of the importance IL-33 plays in clearance of helminth infection, there remains a great deal to be uncovered, as this species specificity necessitates work across the broad body of organisms that exist. The effects of IL-33 on helminth infection is summarized in Figure 2.

## Potential Immunotherapy

Parasitic diseases are responsible for an extensive morbidity and mortality burden across many countries and are caused by wildly different organisms ranging from unicellular protozoans to multicellular arthropods and worms (https://www.cdc.gov/parasites/about.html). There is currently no vaccine approved for human use for any parasitic disease, although the GlaxoSmithKline Biologicals' RTS,S vaccine against malaria has now successfully completed phase III clinical trials (https://www.cdc.gov/malaria/malaria_worldwide/reduction/vaccine.html). Lack of effective preventive measures, combined with poor disease management practices, drug toxicity and a rise in parasitic resistance, have resulted in the high incidence and prevalence of parasitic infections seen in both developing and developed countries alike (Singh et al., 2019). For many years the scientific community has been working on developing novel strategies to prevent, contain and combat parasitic diseases. Investigating the early immune responses to parasitic infections could provide an insight on novel therapeutic targets and approaches (Table 1).

**Table 1:** Summary of the role of IL-33 in parasitic infections.

**Parasite**	**Role of IL-33**	**References**
*Toxoplasma gondii*	T1/ST2^−/−^ BALB/c mice showed increased parasite burden in the brain	Jones et al., 2010
	Susceptible C57BL/6 mice showed increased IL-33 expression correlating with Th2 cytokines in an ocular model of toxoplasmosis	Tong and Lu, 2015; Zhang et al., 2019
	ST2 deficient C57BL/6 mice showed increased survivability in an oral model of toxoplasmosis	Ryffel et al., 2019
*Plasmodium spp*.	C57BL/6 mice show no difference in IL-33 expression 1.5 hours after *P. berghei* infection	Mac-Daniel et al., 2014
	Patients who died from *P. falciparum* infection showed significant IL-33 lung accumulation	Ampawong et al., 2015
	C57BL/6 mice in a *P. bergei* ANKA model demonstrated decreased IL-33 after dexamethasone treatment associated with decreased respiratory distress	dos Santos Ortolan et al., 2018
	IL-33 is significantly elevated in patients under 5 infected by *P. falciparum*	Ayimba et al., 2011
	IL-33 administration to C57/BL6 mice in a *P. berghei* ANKA model demonstrate attenuated cerebral malaria	Besnard et al., 2015
	IL-33 administration increased efficacy of anti-malarial drugs artesunate and chloroquine in a murine model of cerebral malaria	Strangward et al., 2018
	ST2 deficient mice infected with *P. berghei* show reduced cerebral malaria	Palomo et al., 2015
	Reduced inflammatory cytokine expression induced by ST2 deficiency is correlated with improved survival in *P. berghei* mice	Reverchon et al., 2017
	ST2 deficient BALB/c mice demonstrate reduced mortality and hepatocyte damage in a *P. chaubadi* model	Seki et al., 2018
	*P. bergei* infected IL33 deficient mice demonstrate survival and parasitemia similar to wild type mice	Shibui et al., 2016
*Leishmania* spp.	Increased IL-33 is detected in serum from *L. donovani* patients	Rostan et al., 2013; Takele et al., 2016
	ST2 deficient mice demonstrate an ability to control parasite burden and reduced hepatomegaly and splenomegaly in an *L. infantum* model	Khalid et al., 2017
Helminth Infection	Helminth activity causes an increase in IL-33 mRNA expression	Andronicos et al., 2012
	*Hymenolupis diminuta* infection of mice deficient in mast cells have reduced IL-33 associated with increased parasite burden	Hepworth et al., 2012; González et al., 2018
	Mice pre-sensitized to an allergen prior to *Ascaris lumbricoides* infection demonstrate singificantly enhanced IL-33 expression and decreased parasite burden	Gazzinelli-Guimaraes et al., 2019
	IL-33 deficient mice cannot effectively recruit eosinophils or induce goblet cell hyperplasia	Yasuda et al., 2012
	IL-33 deficient mice cannot effectively recruit ILC2 cells resulting in increased parasite burden	Yasuda et al., 2012, 2018
	*Heligmosomoides polygyrus* exosomes inhibit IL-33/ST2 and M2 differentiation	Buck et al., 2014; McSorley et al., 2014; Coakley et al., 2017
	*Ascaris suum* exosomes likely inhibit IL-33	Hansen et al., 2019
	*Fasciola hepatica* exosomes demonstrate an upregulation of IL-33	Finlay et al., 2016

As one of the earliest responses to damage or infection, IL-33 finds itself within a unique niche in host immunity to parasitic infection, where it plays a role in priming and modulating the adaptive immune response. IL-33 has been shown to induce both Th1 or Th2 differentiation depending on the stimuli, and more recently, IL-33 is shown to also influence the behavior and physiology of Th17 and Tregs (Alvarez et al., 2019). Potentially having an impact on such a variety of T cells, this pleiotropic role makes IL-33 an interesting target for pharmaceutical immune-modulation. For example, IL-33 was shown to induce a protective Th2-polarized response during helminth diseases. Th2 cytokines such as IL-5 and IL-13 play a protective role in intestinal helminth infections by inhibiting the adhesion of these parasites to the intestinal lumen (Hasnain et al., 2011). IL-33 signaling was also required for controlling *toxoplasma* infection in the brain and preventing the development of encephalitis (Jones et al., 2010), while absence of IL-33 receptor/ST2 attenuated neutrophilic inflammation and ileitis in an oral model of toxoplasmosis (Ryffel et al., 2019). Additionally, IL-33 was shown to have pro-inflammatory properties during malaria, leading to an exacerbated pathology both in the respiratory and central nervous system (Yasuoka et al., 2011; Fairlie-Clarke et al., 2018). Because of its dual properties, IL-33 could serve as an immune-modulatory target for the early or prophylactic therapies against parasitic infections, where controlled intervention could possibly allow for a more dictated adaptive immune response by the physician.

Interestingly, one application of IL-33 immunomodulation currently being explored is that of helminth therapy: the intentional ingestion of helminth ova or their larvae to induce infection. While not presently widely administered, there are several ongoing and recruiting clinical trials exploring such therapies (NCT02754609, NCT01940757, NCT03565939) with several more having been already completed. Each of these current studies possesses the common theme in that they are investigating how helminth infection is capable of suppressing inflammatory disorders. However, there is a wealth of information suggesting the possibility that helminth therapy could have success as an agent against allergies. A recent study has uncovered that patients infected with *Schistosoma mansoni* present with suppressed immune responses against dust mite allergen. This finding was further associated with increased IL-10, but an inverse correlation with IL-33 (Resende et al., 2018). While it is unrealistic to expect a patient to willingly harbor a helminthic infection to combat a chronic infection such as allergy, there are groups investigating the therapeutic potential of the helminth exosome. Already evidence is suggesting that certain helminth extracellular vesicles are capable of modulating the IL-33/ST2 signaling pathway, which has the capability of suppressing ILC2 accumulation and eosinophilia resulting in an abrogated allergic response (McSorley et al., 2014; Ball et al., 2018). Recent research has even uncovered a specific helminth exosome protein, HpARI, whose immunomodulatory role results in suppression of IL-33 (Osbourn et al., 2017). Moreover, there exists data suggesting that some of these parasite origin vesicles have the capacity to effectively function in potential vaccines (Coakley et al., 2017). Provided these protein isolates can be proven to be both safe and efficacious in human patients, helminth derived products may prove to be powerful immunotherapeutic agents. Already a body of evidence exists indicating that those helminth products which have been tested in human patients are likely to be both safe and effective, while generating a primarily Th2 immune response as would be anticipated (Williams et al., 2017; Capron et al., 2019). However, with such a wide variety of helminth species and exosomes to explore, it is imperative to keep in mind that not all helminths behave the same, and that while those helminths currently studied show promise, it is possible, and even likely, that better helminth therapy candidates exist that are yet to be explored (Sobotková et al., 2019).

Not only does IL-33 play a role in parasitic disease, but implications for IL-33 have also been found in the context of cancer. Previous studies have shown that this cytokine is released by fibroblasts, endothelial and epithelial cells in response to damage or cell death and can aid tumor growth by acting directly on the tumor cells to enhance proliferation and survival and by promoting angiogenesis in the tumor microenvironment. Blood vessel generation facilitates the infiltration of cancer exacerbating cells, including but not limited to, myeloid derived suppressor cells and M2s, upon which IL-33 is able to further exert effect (Afferni et al., 2018). On the other hand, IL-33 also stimulates infiltration of CD8^+^ T cells as well as natural killer cells, crucial for tumor elimination (Gorbacheva and Mitkin, 2019). A better understanding of this differential recruitment of either pro- or anti-inflammatory immune cells in cancer models may help to explain the variable responses seen thus far in IL-33/ST2 malaria research. Additionally, recruitment of such varying immune cell populations is likely to play an important role in *Toxoplasma* infection and clearance, where a balance must be struck between pro-and anti-inflammatory responses to control infection. The net effect of IL-33 on cancer and its progression depends on the type of tumor and its microenvironment. In general, IL-33 as well as ST2 have been implicated in enhanced pathology and progression of colorectal, lung, breast and gastric cancer, as well as melanoma, head and neck squamous cell carcinoma and cholangiocarcinoma (Gorbacheva and Mitkin, 2019; Hong et al., 2019). In terms of hematological malignancies, the effects of IL-33 are controversial as a negative role has been identified for Chronic myelogenous leukemia (CML), while a positive role was shown for Acute myeloid leukemia (AML) (Allegra et al., 2019). A very recent paper by Yue et al. showed that IL-33 stimulates the recruitment of Tregs through the NF-kB/CCL2 pathway, thereby enhancing tumor growth and metastasis in esophageal squamous cell carcinoma (Yue et al., 2019). Another study showed that IL-33 serves a pivotal role in the functional stability of suppressive Tregs in the tumor microenvironment. IL-33 deficient Tregs show attenuated suppressive activity which led to augmented tumor regression (Hatzioannou et al., 2000). A similar positive correlation between IL-33 and Tregs has also been identified in head and neck squamous cell carcinoma, which is further associated with a poor prognosis. ST2 blockade was shown to abrogate the ability for IL-33 to promote Treg activity *in vitro*, however this function has yet to be clarified *in vivo* (Wen et al., 2019). This same ST2 blockade may present itself as an interesting avenue of exploration in the context of leishmaniasis treatment, where down regulation of the T regulatory response and an associated heightened inflammatory immune response has the potential to promote parasite clearance. The involvement of IL-33 in Treg regulation should be further explored in the context of cancer as well as parasitic diseases, as IL-33 immunotherapy is presenting itself as a potential therapeutic option.

Despite IL-33 being one of the earliest responders during infection involved in priming the host immune system toward a Th2 response, its involvement in many parasitic diseases is widely unexplored. A literature search will yield few results for the pathogens mentioned in this review and no results for other parasites notably including trypanosomas, amoebas, trichomonas or ectoparasites. Better understood, this largely unexplored cytokine and its signaling pathways could aid the development of new therapeutics against parasitic, and other conditions.

## Author Contributions

SO contributed to the conception and design of the study. NR, KA, GV, SV, MS, SS and SO wrote sections of the manuscript. All authors contributed to manuscript revision, read and approved the submitted version.

## Conflict of Interest

The authors declare that the research was conducted in the absence of any commercial or financial relationships that could be construed as a potential conflict of interest.
